# Vehicle Headlight Halo Simulation of Presbyopia-Correcting Intraocular Lenses

**DOI:** 10.1167/tvst.12.12.19

**Published:** 2023-12-21

**Authors:** Thomas Kohnen, Sahar A. Nouri, Daniel Carson

**Affiliations:** 1 Department of Ophthalmology, Goethe University, Frankfurt, Germany; 2 Alcon, Inc., Fort Worth, TX, USA

**Keywords:** halo assessment, intraocular lens, bench study

## Abstract

**Purpose:**

This optical bench study was designed to evaluate and compare the halos generated by presbyopia-correcting intraocular lenses (PCIOLs) and monofocal intraocular lenses (IOLs), with or without lens decentration, using an optical bench to simulate the headlight of a distant vehicle in mesopic conditions.

**Methods:**

Halos generated by six nondiffractive and 10 diffractive IOLs with different dioptric add powers were evaluated using a high dynamic range bench system. Halo intensities were compared by assessing the area under the measured intensity profile curve to compute the relative halo magnitude (RHM).

**Results:**

Nondiffractive PCIOLs produced smaller and less intense bench halo images than diffractive ones. RHM measurements ranged from 964 to 1896. Monofocal IOLs produced lower RHM values, whereas diffractive PCIOLs generated higher ones. When decentered by 0.5 mm with respect to the system aperture, more obviously asymmetric halo image profiles were observed in diffractive compared with nondiffractive PCIOLs.

**Conclusions:**

Simulated bench halos of nondiffractive PCIOLs are smaller and less intense than those of diffractive PCIOLs. Additional clinical studies assessing standardized patient-reported outcomes measures are required to correlate these bench results with patient satisfaction.

**Translational Relevance:**

This study contrasts the design-related simulated bench halos of nondiffractive and diffractive PCIOLs, aiming to elucidate their impact on halo perception.

## Introduction

Presbyopia-correcting intraocular lenses (PCIOLs) are a mainstay for both refractive lens exchange and cataract surgery, where they are used to replace the patient's lens or to correct refractive errors. The technology used in PCIOLs undergoes continuous improvement, particularly with the aim to help recipients achieve spectacle independence.^1^ The first PCIOLs were bifocal, providing good quality of vision at near and far distances.^2^ Subsequently, trifocal intraocular lenses (IOLs) were developed, which allowed additional visual restoration at intermediate distance, addressing one of the shortcomings of bifocal IOLs.^2^

More recently, PCIOLs with extended depth of focus (EDoF), also known as EDoF IOLs, have been introduced. The elongated focus prevents the overlapping of near and far images caused by traditional multifocal IOLs, thus minimizing the effect of halo. These IOLs help deliver a continuous range of vision from intermediate to far distances, with some demonstrating improved vision at near distances.^3^^,^^4^

When compared with monofocal IOLs, multifocal IOLs are generally associated with more visual disturbances and a reduced contrast sensitivity,^5^^,^^6^ likely due to the static refractive correction they provide.^7^ After multifocal IOL implantation, patients lose their accommodative capacity and are presented with in-focus and out-of-focus retinal images of the same object, depending on the different foci specific to each IOL.^1^^,^^8^ When viewing a light source in mesopic conditions at distance, the out-of-focus image will be seen as a halo.^8^^,^^9^

In clinical practice, visual disturbances have been assessed using different tools including psychophysical tests,^9^^,^^10^ bespoke questionnaires,^11^ subjective questions during a verbal patient–physician interview,^6^ or a collection of patient-initiated complaints.^12^ A recent review on PCIOLs highlighted the unmet need for a consensus in the clinical evaluation of visual disturbances for both EDoF and multifocal IOLs.^13^

Given the variability of patient-reported outcome measures, objective laboratory methods are desirable for preclinical halo assessments.^13^ In the present study, a high dynamic range (HDR) optical bench system was utilized to evaluate and compare the luminance profile of halo images formed by contemporary PCIOLs and monofocal IOLs at mesopic conditions, using the area under the radial luminance curve to create a relative halo magnitude (RHM).

Previously, laboratory halo measurements were compared to patients’ subjective clinical ratings of halos.^14^ The clinical study showed three pictures representing mild, moderate, or severe halos to patients who had received IOL models, and each patient was asked which one most closely matched the patient's experience. The patient could also select “none.” Plotting the responses of patients with different models of IOL against the laboratory RHM measurements for the same IOL showed good correlation. Therefore, this method provides a reliable quantitative measurement, as well as a relative assessment of different IOL designs.^14^

The American National Standards Institute (ANSI) has issued standard Z80.35 for EDoF IOLs,^15^^,^^16^ which includes a laboratory test for unwanted visual effects, including halo. The optical bench used in this study conforms to standard Z80.35, and data were collected according to the test in the standard. The standard requires collecting images of a light source at the distance focus of the IOL. Test results are a printed image, a radial light intensity graph with visual angle on the horizontal axis, and the logarithmic value of the light intensity on the vertical axis.

Standard Z80.35 requires that the image data for an EDoF lens be compared to data from a monofocal and a multifocal IOL but does not include a quantitative metric for evaluation of the image data. In the current study, the area under the radial light intensity graph is computed to create a single metric for halo image size and intensity—the RHM.

The human visual system can perceive very bright and very dim images at the same time, so that a person driving a car at night frequently sees a range of 4.5 log units of intensity.^17^ In order to capture a realistic dynamic range in the halo images, the HDR camera system used in this study can capture 6 log units of intensity in the same image. This high dynamic range ensures that the radial intensity plot (and the RHM value computed from it) captures the full range of intensities seen by a human observer.

## Methods

### Bench Description

**Figure 1.**
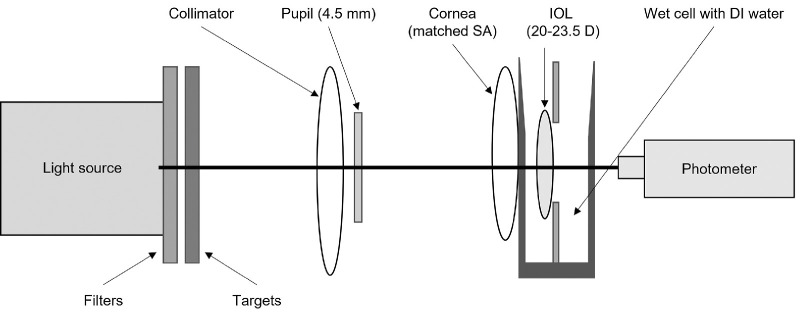
Schematic of halo bench settings, components, and layout.^1^^,^^2^ DI, deionized.

This study used the HDR Halo System (Alcon, Inc., Fort Worth, TX). As illustrated in Figure 1, it consisted of the following components:

1. A 16-bit, WP-103 HDR photometric camera with a cooled 2.76-megapixel imaging photometer (Westboro Photonics, Ottawa, ON, Canada) attached to a DIN length tube and a 4× Plan Achromatic microscope objective lens
2. Model eye using an aspherized convex-plano artificial cornea
3. Wet cell filled with deionized water (set at room temperature)
4. An artificial pupil coupled to a collimator, equivalent to 4.5-mm diameter at the IOL plane (a 4.5-mm pupil size simulates mesopic conditions and conforms to ANSI Z80.35-2018)
5. Wavelength filter to reproduce photopic spectral response of retina (the luminance measured appeared pale green)
6. Incandescent light source of a 150-W bulb illuminating a pinhole target

The optics of the system simulate a 100-mm-diameter headlight at 30 meters at mesopic conditions, with a total field of view of 6°. The size of the field of view was selected to be able to capture the halo images from all IOLs tested.

### Intraocular Lenses

Six nondiffractive and 10 diffractive IOLs with different dioptric add powers were tested, using one sample of every IOL (Table). The IOLs were tested at their best distance focus with a 4× objective, and categorized as monofocal, monofocal plus, EDoF wavefront shaping, EDoF progressive aspheric IOLs (nondiffractive), or EDoF diffractive, bifocal, trifocal, or EDoF/multifocal (diffractive) IOLs. All IOLs, except monofocal lenses, were defined as PCIOLs according to the manufacturer's directions for use.

Although the choice of distance power tested was limited by the availability of IOLs, all tested samples were either 20.0 or 21.0 diopter (D), except for two IOLs: one at 23 D and another at 23.5 D. Increasing IOL power results in a smaller image, but a 1-D difference reduces image size by only 1.6%, which should not affect the comparison of the various IOL models tested in this study. For the highest power IOL tested, 23.5 D, the image size was 3.4% less than if it had been a 21-D IOL of the same model.

**Table.** Optical Characteristics of Intraocular Lenses Tested^25^^,^^26^^,^^40^^–^^54^

IOL (Model), Manufacturer	Category	Optical Principle	Distance Power (D)	Intermediate/Near Add Powers (D)
Clareon (SY60WF), Alcon	Monofocal	Nondiffractive	21	–/–
TECNIS Monofocal 1-Piece (ZCB00), Johnson & Johnson	Monofocal	Nondiffractive	20	–/–
AcrySof Single-Piece (SA60AT), Alcon	Monofocal	Nondiffractive	21	–/–
TECNIS Eyhance (ICB00), Johnson & Johnson	Monofocal plus	Nondiffractive	20	–/–
Clareon Vivity (CNWET0), Alcon	EDoF	Wavefront shaping	20	–/–
MINI WELL (Z7560CZ), SIFI	EDoF	Progressive aspheric	21	–/–
AcrySof IQ ReSTOR +2.5 D (SV25T0), Alcon	Bifocal	Diffractive	21	–/2.5
PhysIOL FineVision (MICRO F), BVI	Trifocal	Diffractive	20	1.75/3.5
TECNIS Multifocal (ZLB00), Johnson & Johnson	Bifocal	Diffractive	20	–/3.25
Clareon PanOptix (CNWTT0), Alcon	Trifocal	Diffractive	20	2.2/3.25
AT LISA tri (839MP), Carl Zeiss Meditec	Trifocal	Diffractive	21	1.66/3.33
AcrySof IQ ReSTOR +3.0 D (SN6AD1), Alcon	Bifocal	Diffractive	21	–/3.25
PhysIOL FineVision Triumf (POD L GF), BVI	Trifocal	Diffractive	23	1.75/3.5
AT LARA (829MP), Carl Zeiss Meditec	EDoF	Diffractive	23.5	0.95/1.9
TECNIS Symfony OptiBlue (ZXR00V), Johnson & Johnson	EDoF	Diffractive	21	NR^a^
TECNIS Synergy (ZFR00V), Johnson & Johnson	EDoF/multifocal	Diffractive	20	NR^a^

NR, not reported.

a Powers were not disclosed by the manufacturer.

### Data Collection

All IOLs were mounted in the model eye of the HDR Halo System, immersed in the wet cell, and placed at an axial position of 26.5-mm vergence distance. Artificial corneas with several different amounts of spherical aberration (SA) were available, and, for each IOL, a cornea was selected that best matched the designed SA of the tested IOL. The best distance focus of each IOL was determined by moving the camera position with a computer-controlled stage (Thorlabs, Inc., Newton, NJ), while visually observing a 1951 United States Air Force (USAF) target image. The target was then changed to the pinhole, and images were obtained using Photometrica 7.5 (Westboro Photonics, Ottawa, ON, Canada). For selected IOLs, which were representative of diffractive and nondiffractive PCIOLs from multiple manufacturers, measurements were repeated with a 0.5-mm decentration relative to the artificial pupil at the best distance foci.

The use of matching corneas for each IOL results in halo images that include little or no SA, a condition that does not represent typical clinical results. Optical theory shows that the primary effect of residual SA is to increase the size of the central image. Our results of testing an IOL with the corneal SA mismatched by 0.1 µm increased the RHM value by 6.4% compared to using the SA-matched cornea (data on file, 2020). Therefore, fully correcting the IOL SA was judged to be the best method of obtaining RHM values that could be compared among IOL models with different SAs.

### Data Analysis

The luminance of the images obtained was analyzed with Photometrica 6.5 and compared using the logarithmic representation of images to facilitate the display of the halos, which are much dimmer than the central image. At the beginning of the testing, the intensity at the center of the image from a monofocal IOL was set to a level just below saturation and stayed at that level for all samples. This method of setting intensity provided consistent results. The ALOHA software (Alcon) computed the RHM of every logarithmic image, calculated from the average intensity cross-section to an extent of 3° starting at the center of the pinhole image along 8 lines 45° apart. The RHM calculation is given in arbitrary units, as the vertical axis is normalized, with the peak luminance values at zero normalized to 1. The brightness of each image was increased 20% using a photo editor to further emphasize the halos.

**Figure 2.**
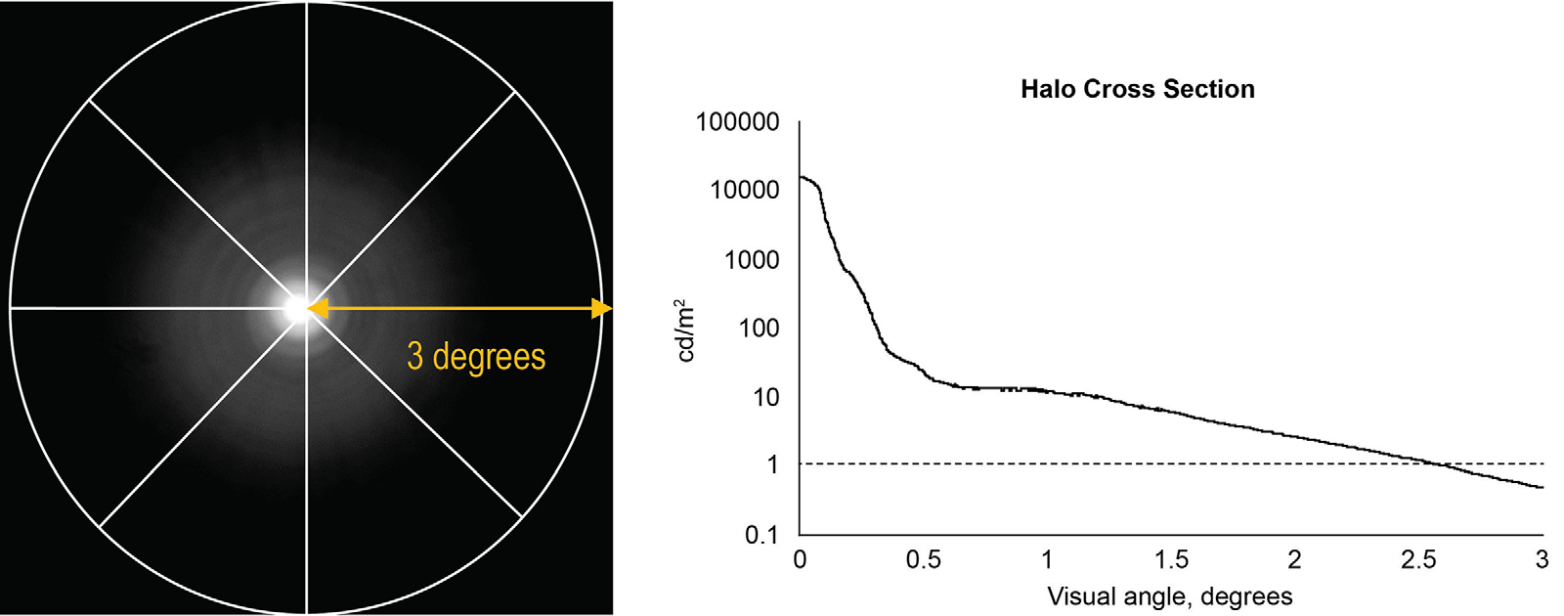
Eight radial profiles of a bench halo image (*left*) are averaged over a 6° field of view, and the RHM is calculated from the area under the profile curve (*right*). The example shown is IOL model ZFR00V.

The 20% brightness adjustment was done to aid visualization of the presented figures, but it had no effect on the RHM calculation, which was done using the objective candela per square meter (cd/m^2^) values in the image data. An example of an RHM calculation of a diffractive halo profile is shown in Figure 2. Higher RHM values indicate greater halo effects. Qualitative assessment of halo asymmetry was used for decentered analysis, as the asymmetry can cause the RHM value to decrease, which could complicate interpretation of quantitative analysis. No statistical analyses were performed for this study, as it was not possible to obtain enough samples of each IOL model to power a statistical analysis of the measurements. The approach of using one IOL model of each type tested and no statistical analysis has been used previously in the literature.^18^^,^^19^ Regulatory agencies hold manufacturers of implanted devices to consistent quality standards; hence, we do not expect large sample-to-sample variation in bench test results of IOLs. Separately, the testing laboratory used in the present study has established the repeatability of the RHM measurement by a Gage repeatability and reproducibility (GR&R) study and has shown that the RHM reliably differentiates among IOLs of different designs.^14^

## Results

### Centered Analysis

**Figure 3.**
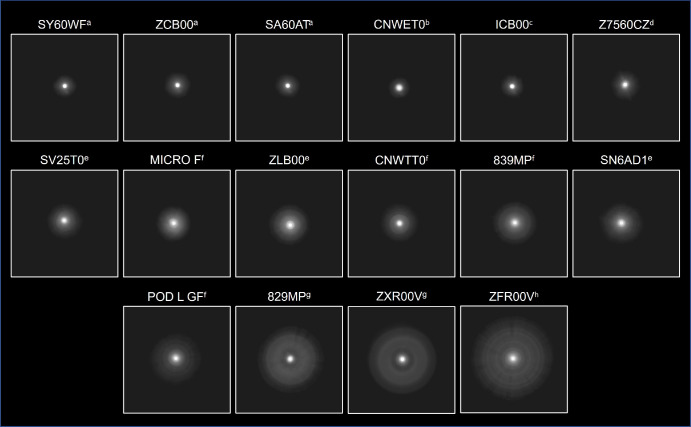
Bench-simulated halo images. To save space, from the measured total field of view of 6°, the field of view shown is 4.5° for each image. ^a^Monofocal; ^b^EDoF wavefront shaping; ^c^monofocal plus; ^d^EDoF progressive aspheric; ^e^bifocal; ^f^trifocal; ^g^EDoF diffractive; ^h^EDoF/multifocal diffractive.

**Figure 4.**
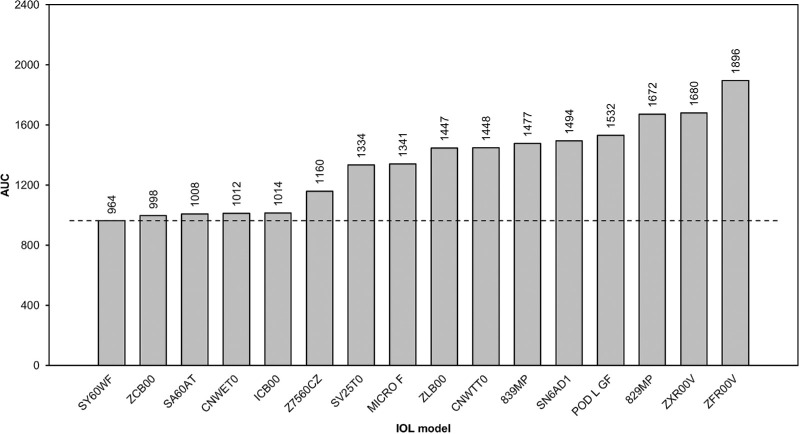
RHM of bench halo images, integrated over 0° to 6° field of view (FoV).

All IOLs were successfully assessed. Halo images and their corresponding RHM values are presented in Figures 3 and 4, respectively. The images in Figure 3 are ordered by increasing RHM values and IOL category. The images in Figure 3 represent a 4.5° field of view. Monofocal IOLs included SY60WF (Alcon), ZCB00 (Johnson & Johnson, New Brunswick, NJ), and SA60AT (Alcon); these presented lower RHM values compared with monofocal plus lenses and PCIOLs, suggesting smaller and/or less intense halos. The RHM values were 964, 998, and 1008 for SY60WF, ZCB00, and SA60AT, respectively.

The wavefront shaping EDoF CNWET0 (Alcon) and progressive aspheric Z7560CZ (SIFI SpA, Catania, Italy) PCIOLs displayed halo images most similar to those of monofocals. The RHM values were 1012 for CNWET0 and 1160 for Z7560CZ. With an RHM of 1014, ICB00 (Johnson & Johnson), the only monofocal plus IOL included in this study, had a halo image greater than CNWET0 and less than Z7560CZ.

RHM values for bifocal and trifocal lenses ranged from 1334 for SV25T0 (Alcon) to 1532 for POD L GF (BVI Medical, Waltham, MA). SV25T0 and MICRO F (BVI Medical) displayed relatively small halo images with RHM values between EDoF and diffractive PCIOLs. EDoF or multifocal diffractive IOLs had greater halo images (RHM ≥ 1400) compared with all other lenses tested. The 829MP (Carl Zeiss Meditec, Oberkochen, Germany) sample was higher power than the rest, indicating that the RHM of a 21-D sample should be somewhat higher than 1672, but it would still be one of the three largest halo images, fitting between POD L GF and ZFR00V (Johnson & Johnson); ZFR00V exhibited the highest halo image size and intensity (RHM = 1896).

### Decentered Analysis

**Figure 5.**
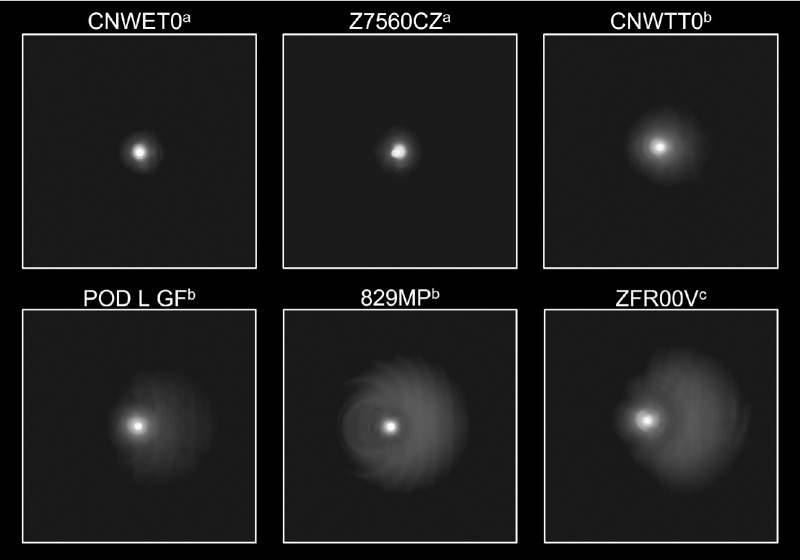
Halo images, 6° field of view, decentered by 0.5 mm. ^a^EDoF nondiffractive; ^b^trifocal; ^c^EDoF/multifocal diffractive.

The EDoF wavefront-shaping, EDoF progressive aspheric, three trifocal, and one EDoF/multifocal diffractive lenses were used for the 0.5-mm decentration analysis, as shown in Figure 5. Although decentered by 0.5 mm with respect to the system pupil, for CNWET0 and Z7560CZ the asymmetry of the halo image profile to the central spot was small and difficult to see. With regard to trifocal or EDoF/multifocal diffractive lenses, ZFR00V produced the most asymmetric halo image after decentration, followed by 829MP, POD L GF, and CNWTT0 (Alcon).

## Discussion

Optical bench evaluations, such as spread point function and modulation transfer function,^8^^,^^20^^,^^21^ have been proposed to provide valuable information on IOL performance with regard to image quality and photopic phenomena. In the present study, a novel HDR method was used to evaluate and compare the optical performance of 12 PCIOLs and four monofocal IOLs concerning halo formation using the RHM metric. This method was chosen as it has been previously shown to be associated with patient-reported visual disturbances in earlier IOL models.^14^ The test simulated the headlight of a vehicle in mesopic conditions, representing a relevant visual situation associated with the perception of halos.^9^

Patient dissatisfaction due to the occurrence of visual disturbances inherent in IOL design can be challenging to prevent or manage.^22^ Although qualitative assessment of halos via patient questionnaires is useful, RHM measurement provides a reliable quantitative assessment, aiding personalized IOL selection with the aim to further improve patient outcomes and satisfaction.

The RHM value does not account for structure in the halos from IOLs; however, good correlation of RHM to patient reports of objectionable halo has previously been reported.^14^ The two-dimensional images are useful in visualizing halo details not accounted for by the RHM value (Fig. 3).

The HDR optical system used in this study revealed that smaller and less intense halos were produced by nondiffractive versus diffractive PCIOLs. The halo image produced by CNWTT0 was less intense than that of ZXR00V (Johnson & Johnson), in line with a recent study evaluating older models of these lenses (TFNT00, Alcon; ZXR00, Johnson & Johnson).^23^

CNWET0 produced the lowest RHM value of the EDoF IOLs examined. CNWET0 and ZXR00V are currently the only EDoF IOLs tested in this study with available clinical assessment in line with the ANSI criteria (ANSI Z80.35-2018).^15^^,^^24^^–^^26^ These categorize IOLs as EDoF based on minimum clinical performance, assessed by testing visual acuity for distance and intermediate vision, as well as defocus curve.^15^^,^^16^ The criteria also recommend the laboratory and subjective assessment of limiting visual disturbances, but do not indicate a maximum acceptable level.^15^^,^^16^

Notably, CNWET0 and Z7560CZ displayed an RHM value similar to the monofocal IOLs SY60WF, ZCB00 and ICB00. CNWET0 is characterized by wavefront-shaping technology located on the anterior surface and consists of two elements that work synergistically to focus all incoming light within an effective EDoF channel reaching the retina.^27^ In contrast, Z7560CZ presents three concentric zones: a central zone with positive SA, a middle zone with negative SA, and an outer monofocal aspheric zone.^28^

Previous testing on CNWET0 demonstrated that the lens has the capability to provide good vision from distance to functional near, further improving the distance image quality at a 4.5-mm versus 3-mm system aperture and reflecting performance in different light conditions.^21^ The monofocal-like halo image of CNWET0 obtained through bench simulation is in line with qualitative patient-reported outcomes measures collected in previous studies including two randomized controlled trials.^24^^,^^25^^,^^29^^,^^30^ In addition, two studies suggest that Z7560CZ has better optical quality than 839MP (Carl Zeiss Meditec), MICRO F, and ZXR00 at distance, using an aperture of 4.5 mm.^31^^,^^32^ These findings suggest that both CNWET0 and Z7560CZ may represent an appropriate alternative to a standard monofocal IOL for patients undergoing cataract or refractive lens change.

In terms of decentration, pseudophakos is known to produce glare, halos, and other visual aberrations.^33^ A study that set to replicate the optical performance of pseudophakic eyes with various IOL surface designs at different orientations of IOL misalignment (0.4-mm decentration, 7° tilt, and angle κ = 0.5 mm) found a significant negative impact on postoperative quality of vision.^34^

IOL decentration represents a concern for monofocal IOLs and even more so with aberration-correcting IOLs.^35^^–^^37^ Given that there is a clear link between lens decentration and halos, it is important that both are measured when carrying out a halo simulation bench study to establish a complete profile for each PCIOL. When decentered by 0.5 mm with respect to the system pupil, POD L GF, 829MP, ZFR00V, and CNWTT0 showed more obvious asymmetry in the halo images than CNWET0 and Z7560CZ. This analysis suggests a potential value of implanting nondiffractive IOLs when a lens displacement is likely to occur, such as in eyes with long axial length, over-large capsulorhexis, or thick crystalline lens.^38^

Differences in optical lens properties (e.g., diffractive pattern, apodization, lens asphericity) may have contributed to the observed variability in halo image size and intensity among the IOLs. Halos produced by multifocal IOLs have been previously shown to vary in size and intensity according to the lens add power.^8^ When comparing the halo image profiles of SV25T0 and SN6AD1 (add powers of +2.5 D and +3.0 D, respectively; Alcon), the former originated a smaller halo effect, resulting in a lower RHM value (difference of 160). As for the other diffractive IOLs tested, add powers did not appear to be associated with halo image intensity, possibly because of other physical characteristics such as apodization, achromatic structure, step height, and/or number of rings.

In clinical practice, patients’ quality of vision using aspheric lenses is usually optimized through two approaches: surgeons may elect either to compensate the positive SA of the cornea using IOLs with negative SA or to implant “aberration-neutral” IOLs designed to perfectly refract a collimated light beam onto the focal point.^39^ The present study included IOLs with different degrees of negative SA, for which the corneal SA of the model eye was adjusted.

The present method offers a strong baseline for surgeons to better understand and interpret the optical performance of currently available PCIOLs in terms of halo propensity. Efforts were made to assess mid–power-ranged IOLs from various manufacturers, with a variety of optical designs. Nevertheless, the inclusion of IOLs in this study was dependent on their availability at the time of the study.

In conclusion, this study used a bench-based measurement approach to assess the visual performance of currently available PCIOLs with regard to halo formation, with the eventual aim to support preoperative lens selection and optimize patient satisfaction. Simulated bench halo images of nondiffractive PCIOLs were smaller and less intense than diffractive PCIOLs when tested under mesopic conditions. Given that halos are only one of several factors contributing to patient satisfaction, further studies are required to understand how these bench results translate into patient experience and whether they can be reproduced using other simulation tests.
